# Visualization and quantification of deformation behavior of clopidogrel bisulfate polymorphs during tableting

**DOI:** 10.1038/srep21770

**Published:** 2016-02-25

**Authors:** Xian-Zhen Yin, Li Wu, Ying Li, Tao Guo, Hai-Yan Li, Ti-Qiao Xiao, Peter York, Ashwini Nangia, Shuang-Ying Gui, Ji-Wen Zhang

**Affiliations:** 1 Center for Drug Delivery System, Shanghai Institute of Materia Medica, Chinese Academy of Sciences, Shanghai 201210, China; 2 Institute of Pharmaceutical Innovation, University of Bradford, Bradford, West Yorkshire BD7 1DP, United Kingdom; 3 School of Life Sciences, Jilin University, Changchun 130012, China; 4 Anhui University of Chinese Medicine, Hefei 230038, China; 5 Shanghai Synchrotron Radiation Facility, Shanghai Institute of Applied Physics, Chinese Academy of Sciences, Shanghai 201204, China; 6 School of Chemistry, University of Hyderabad, Hyderabad 500046, India

## Abstract

The deformation behavior of particles under pressure dominates the mechanical properties of solid dosage forms. In this study, the *in situ* 3D deformation of two polymorphs (I and II) of clopidogrel bisulfate (CLP) was determined to illustrate pressure distribution profiles within the tablet by the deformation of the crystalline particles for the first time. Synchrotron radiation X-ray computed microtomography (SR-μCT) was utilized to visualize and quantify the morphology of thousands crystalline particles of CLP I and CLP II before and after compression. As a result, the deformation was examined across scale dimensions from microns to the size of the final dosage form. Three dimensional parameters such as volume, sphericity, oblate and prolate of individual particle and distributions were computed and analyzed for quantitative comparison to CLP I and CLP II. The different degrees of deformation under the same compression conditions of CLP I and CLP II were observed and characterized quantitatively. The map of deformation degrees within the tablet illustrated the heterogeneous pressure distribution in various regions of the compacted tablet. In conclusion, the polymorph deformation behaviors demonstrated by SR-μCT quantitative structure analysis deepen understanding of tableting across dimensions from microns to millimeters for the macrostrcuture of tablet.

Powder compression represents an essential unit operation in the pharmaceutical industry since the compressed tablet represents the most common solid dosage unit used throughout the world for the delivery of medicines to patients. Mechanical properties, such as compactability, compressibility, elasticity, plasticity, and brittleness of the excipients and drugs as powders, dictate how formulations will behave during tablet processing and perform as drug delivery systems. The physicochemical and solid state properties of powders, such as their polymorphism, moisture content, particle shape, particle size, and surface roughness, affect the mechanical properties of tablets formed from these constituent powders. Therefore, understanding the potential influence of these parameters on the properties of the products during and after compaction is important^1^.

Many solid pharmaceuticals exhibit polymorphism resulting from the possibility of forming at least two crystalline arrangements of molecules in the crystal lattice^2^,^3^. Generally, the polymorphs often exhibit different solid-state properties, density, habits, refractive index, melting properties, solubility, dissolution rate, and mechanical properties^4^. Moreover, under specific environmental conditions, such as pressure and temperature, as well as chemical purity the structure of polymorphs represents the packing of the molecules in the crystal lattice, which directs the solid-state properties of the crystals and also influences the compaction behavior of powders^5^.

During compaction, particles firstly rearrange at the initial stage of compression and then deformation takes place during consolidation. It predominantly depends on the mechanical properties, the deformation behavior of powder under stress may be elastic, plastic, brittle fracture or a combination. Elastic deformation is time independent, reversible and does not continuously influence tablet strength as the residual stresses during the decompression phase. However, plastic deformation is an irreversible process that contributes to the formation of particulate bonds that generate the tablet strength^6^. During the direct compaction process, the density distribution inside the tablets is often heterogeneous due to the inter particle friction and die wall friction. Since the shape of tablets used in pharmaceutical industry varies between flat-faced cylindrical tablets to more complex geometries with embossing, density variations in pharmaceutical tablets may be important and affect the compact mechanical properties^7^ as well as leading to non-uniformity of three dimension (3D) internal structure between individual tablets and consequently potential non-uniform drug release patterns. Density distribution has been investigated using NMR tomography^8^ and autoradiography^9^. Recently, X-ray microtomography has been used successfully in pharmaceutical development studies^10^.

Recently, the relationships between the mechanical properties, deformation behavior and the molecule structure of the polymorphs are gradually becoming better understood. For example, the relationship between crystal structure and the mechanical properties of ranitidine hydrochloride polymorphs has been investigated. Powder X-ray diffraction (PXRD), differential scanning calorimetry (DSC), and optical and polarized microscopy have been combined with compressibility plots and a Heckel analysis to confirm the greater plastic deformation of form II over form I. The Heckel equation provides a method for transforming a parametric view of the force and the displacement data to a linear relationship for the materials undergoing compaction^11^. While studying the compression behavior of orthorhombic paracetamol crystals, the resulting tablets were broken into small pieces, and images of compressed crystals were obtained using scanning electron microscopy (SEM)^12^. However this approach has several disadvantages. The analytical method destroys the integrity of the tablet and might damages the structure of the individual crystal particles to some extent during tablet breakage. Moreover, SEM is not an *in situ* measurement and is unable to reflect the deformation behavior of the crystals during and after the compaction process. Other current methods use various mechanical parameters include Young’s modulus, Poisson’s ratio, yield stress, and fracture toughness to reflect the deformation characteristics of powder, which are difficult to obtain a high-resolution visualization of the *in situ* behavior of individual crystals within the tablets. In addition, methods such as SEM that are used to probe the shape of a limited number of crystal particles do not provide a statistical sampling of all of the crystal particles in the intact tablets.

Synchrotron radiation X-ray computed microtomography (SR-μCT) uses synchrotron generated X-ray with high photon flux and polarization as a powerful non-invasive tool to directly reveal the three-dimensional structure of various objects at a high spatial resolution^13^,^14^. SR-μCT has been applied to various fields, mainly for investigating materials and biological samples, and geological or paleontological samples to a lesser extent^15^,^16^,^17^. Within pharmaceutical materials science and drug delivery research, SR-μCT has been used to visualize and quantify the microstructure of particles and the distribution of chemical composition for individual particles and their assemblies^18^,^19^,^20^. SR-μCT has also been used to distinguish clopidogrel bisulfate (I and II) in combination with multilayer perceptron. The polymorphs of the drugs could be identified and predicted through the numerical description of the 3D morphology^21^. However, to our knowledge, the visualization of the deformation behavior of crystals *in situ* has never been reported as measured by SR-μCT.

In this study, the deformation behavior of two polymorphs of clopidogrel bisulfate (CLP I and CLP II) was investigated by SR-μCT. CLP is a pharmaceutical compound with a novel mechanism of action for the reducing atherosclerotic events. Six different polymorphs are known for CLP. However, only CLP I and CLP II are used as drugs that are administered in solid dosage forms^22^,^23^. As reported, these two polymorphs of CLP were investigated for in-die and out-of-die compaction behavior using CTC profile, Heckel, and Walker equations, and the difference in compaction behavior was explained from the molecular structure^24^. This investigation uses SR-μCT to obtain the individual particle characteristics of CLP polymorphs with an *in situ* 3D view, as well as measuring the deformation behavior of the two crystal forms without destroying the integrity of the tablets and correlating the result with reported mechanical properties. Therefore, our study aims to develop a characterization method for SR-μCT that can determine the *in situ* deformation behavior of the CLP polymorphs after compaction and to analyze the relationship between the polymorphic structures and mechanical behavior during tableting.

## Results and Discussion

### Powder X-ray diffraction of CLP polymorphs

**Figure 1:**
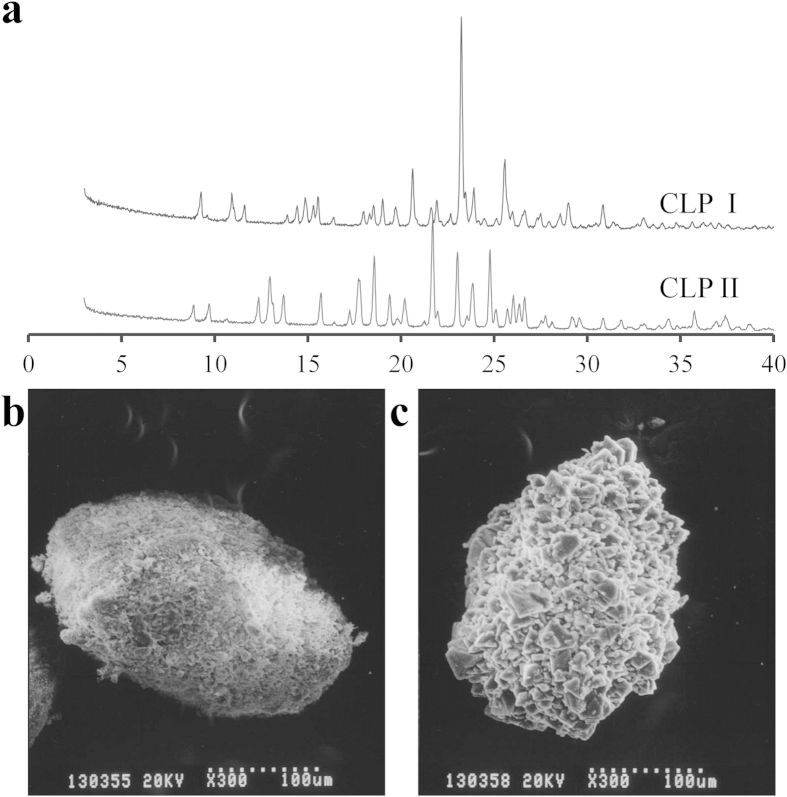
PXRD and SEM of CLP I and II crystal particles. Powder X-ray diffraction overlay of CLP I and II (**a**). SEM (300 × ) of CLP I (**b**) and II (**c**).

The results of the PXRD measurements were consistent with those reported previously (Fig. 1) showing PXRD patterns of both CLP I and CLP II^24^. There were clear differences in the X-ray diffraction peaks for CLP I and II polymorphs. The region 12–13° 2θ was characteristic for polymorph II while the peak at 21° signified form I, without any overlap of peaks from the other polymorph in these regions.

### Scanning electron microscopy

SEM was employed to characterize the surface morphology of the CLP polymorphs. The samples were fixed on the objective table and coated with aurum before observation. The morphological differences were visible between CLP I and CLP II. The surface morphology of CLP II was more rough than CLP I. In the images magnified 300 times, (b) and (c) in Fig. 1 showed that a quite large number of crystals combined into a larger polycrystal. The subunits of CLP I particles were all significantly smaller than those of CLP II, indicating that CLP I crystals were more densely packed compared to those of CLP II, and the larger subunit size of CLP II introduced a loose structure of the accumulated particles due to larger pore space between the particles. Therefore, the particles of CLP II were more easily damaged than those of CLP I during compaction or extrusion.

### Structure of CLP polymorphs particles measured by SR-μCT

**Figure 2:**
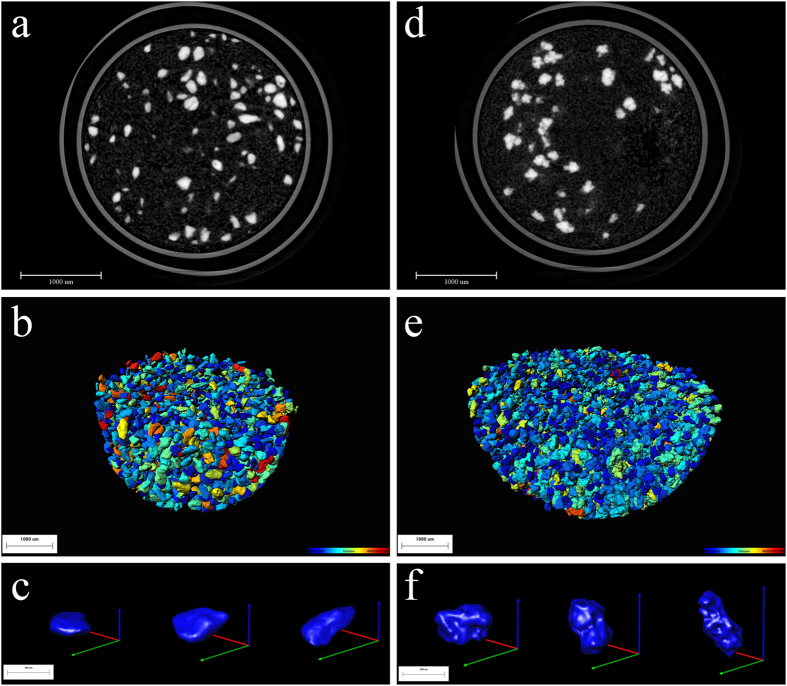
Monochrome 2D slices and 3D morphology of CLP I and II crystal particles. 2D slices of the samples contain CLP particles diluted with PVP/VA (**a**,**d**), 3D images of crystal particles in the capsule after extraction and construction (**b**,**e**), and the randomly selected individual CLP I (**c**) and CLP II (**f**). The color gradients reflect particle volume, ranging from about 8.0E + 4 μm^3^ (dark blue) to 4.0E + 7 μm^3^ (red).

CLP particles were distinguished from the excipients due to the noise reduction and phase retrieval. The gray value of the background was close to 0 for the excipients and the wall of the capsule as container for the powders was between 80 and 120, and the CLP particles gave a gray value above 150. Two dimensional (2D) slices of the crystal particles are shown in Fig. 2; the bright areas represent CLP particles and the PVP/VA are illustrated in gray.

Based on the analysis of gray values, all of the crystal particles in the samples were extracted. Afterward, highly resolved tomographic images of CLP I and CLP II with high quality phase contrast were derived for each single particle after 3D reconstructions (Fig. 2b,e). For the two samples, all of the crystal particles were included in the analysis, and individual particles were assigned a color according to the volume of the individual particles. The morphological structure of the CLP I and CLP II particles were clearly different. Both crystal forms were irregular but the surface topography of CLP II showed greater roughness than CLP I (Fig. 2c,f).

### Visualization of the crystal deformation behavior in tablets by SR-μCT

**Figure 3:**
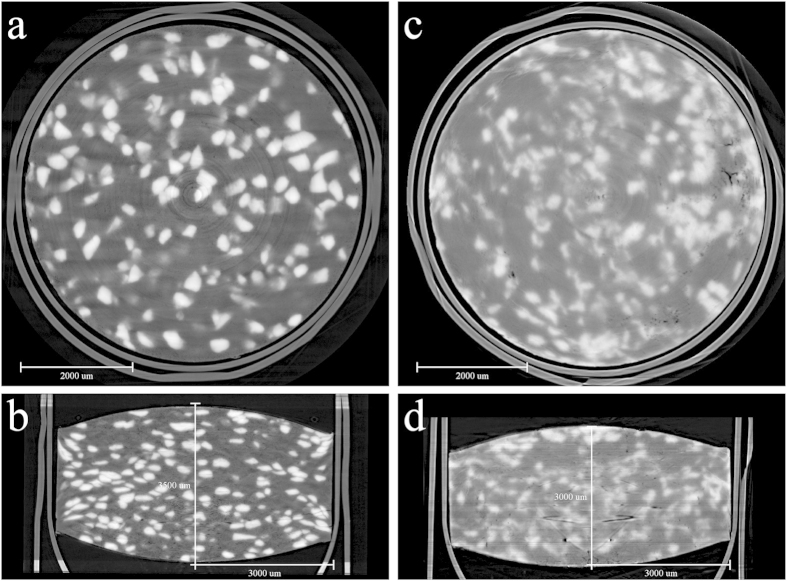
Monochrome 2D slices of CLP tablets. CLP I (**a**,**b**) and CLP II (**c**,**d**). (**a**,**c**) and (**b**,**d**) are planes from the different angles (the outside border is the shell of the gelatin capsule, the container to fix the tablet on the objective table).

Tablets containing hundreds of pure particles of CLP I or CLP II diluted with PVP/VA were put into individual capsules with an internal diameter identical to that of the tablets. From the SR-μCT scan and the analysis of phase contrast extraction by X-TRACT, 2D slices were obtained (Fig. 3), showing the individual crystal particles within the tablet distinguished from the PVP/VA by the difference in the gray level, as discussed above. Compared to the powder samples in Fig. 2, the CLP crystal particles showed a change in shape as a result of the compaction process; specifically, the particles of CLP I exhibited a flatter morphology, while many CLP II particles were fractured into sized particles.

Both of the two tablets contained the same weight of CLP particles and excipients, compacted under the same conditions, and the same compression ratio were obtained by compaction. But the final size, especially the thickness of the tablet was significantly different, namely, about 3,500 μm for CLP I and 3,000 μm for CLP II. The tablet of CLP I bounced back about 17% during the compaction when the pressure was released as the upper punch moved, but CLP II barely changed.

**Figure 4:**
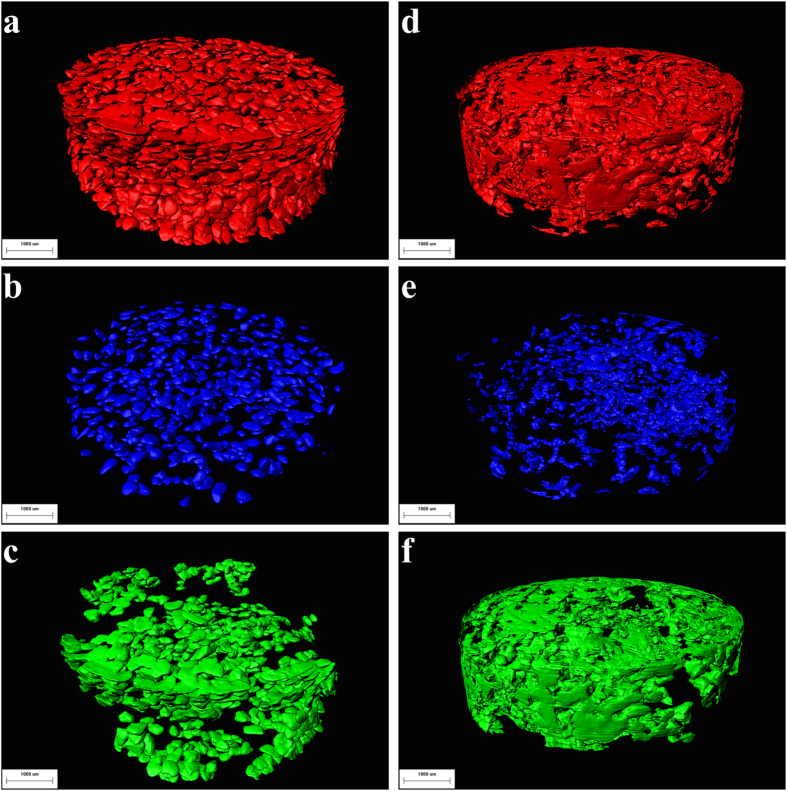
3D models of CLP particles in tablets. Left insets represent CLP I particles, right insets represent CLP II particles, (**a**,**d**) are the 3D models of all particles in tablets, (**b**,**e**) are the 3D models of unattached particle clusters in the tablets, (**c**,**f**) are the 3D models of agglomerated particles in tablets. The scale bar is 1,000 μm.

All the 2D slices were processed by VGStudio Max and Image Pro Analyzer 3D software, and the reconstructed 3D images of tablets were obtained. Figure 4 shows the 3D morphological images of all particles within the tablet samples. In the surface regions of the tablets, the CLP I and CLP II particles exhibited large changes in morphology. The CLP I particles were flattened, while most of the CLP II particles were fragmented and lost their original shape. In the lower density regions, the CLP I particles remained well separated from each other and changed slightly in shape, while the CLP II particles fragmented, although less so close to the die wall.

By deleting particles that retained their original shape and could be distinguished from the other drug particles after tablets formation from the 3D-structure analysis, the reconstructed images of the deformed and fragmented particles in the tablets were obtained (Fig. 4). The images reveal that the particles underwent extensive deformation of the surfaces of the tablets. The shapes of the particles were flattened and combined into agglomerates under pressure, particularly the particles of CLP II that created large agglomerates. Therefore, images prepared by SR-μCT clearly visualized the consequences of the different deformation behavior of CLP I and CLP II. Although some particles of CLP I combined to form large agglomerates, it was still possible to identify their characteristic shape. In contrast, particles of CLP II were fragmented and compressed into a connected framework. It was difficult to identify their original morphology.

**Figure 5:**
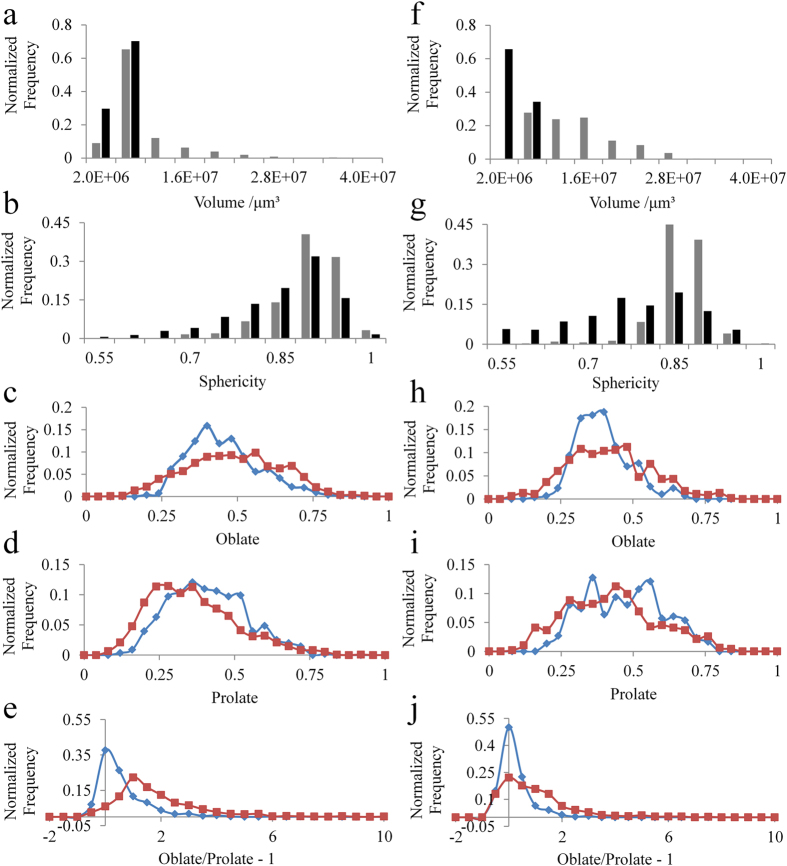
3D quantitative parameters of CLP I and CLP II before and after compression. Particle volume and sphericity distribution changes during tableting of CLP I (**a**,**b**) and CLP II (**f**,**g**). (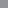 show the normalized volume/sphericity frequency of particles before tableting, ■show the normalized volume/sphericity frequency of particles in tablets after tableting.) The ellipsoid parameters distribution of crystal particles before and after compaction (**c**,**h)**. for oblate, (**d**,**i)**. for prolate and (**e**,**j**). for oblate/prolate-1). (■before compaction, ◆after compaction). The left represents CLP I, the right represents CLP II.

The drug particles that retained their original shape and remained separated from each other after compaction were distributed around the center of the tablets (Fig. 4). The degree of deformation for the particles separated from each other was markedly smaller than that for particles at the surface of the tablets. These particles were extruded under different applied pressures. In general, for curved specimens, the highest density regions tended to be close to the die wall, while the lower density regions were located in the center of the tablets^25^. The different deformation behaviors of CLP I and CLP II were obtained in these images of Fig. 5; each particle of CLP I was scattered in the regions and kept its original shape, while the particles of CLP II were combined with each other. CLP II was more easily crushed than CLP I.

**Figure 6:**
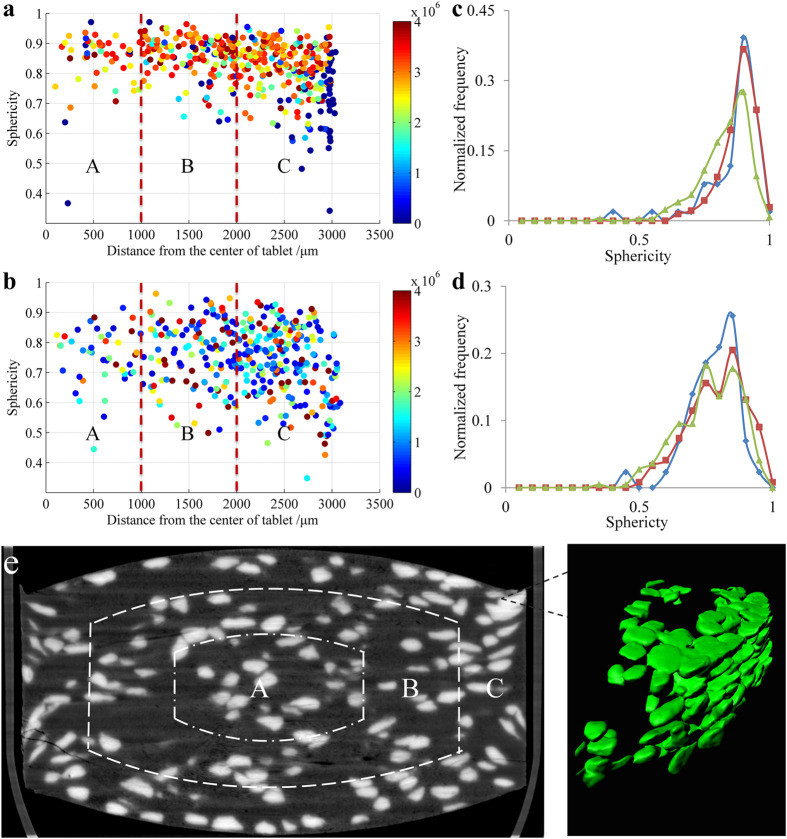
Heterogeneous pressure distribution within the tablets. Particle position (**a**,**b)**. distance from the center of the tablet) and the sphericity colored with volume (Section A: intermediate pressure, Section B: low pressure, Section C: high pressure. The chromatic stripes on the right side of the axis represent the volume (μm^3^), (**a**). CLP I, (**b**). CLP II); (**c**,**d**). Sphericity distributions of CLP I and CLP II in different parts of a tablet. (**c**). CLP I, (**d**). CLP II, ■Section A (intermediate pressure), ◆Section B (low pressure), ▲Section C (high pressure)). (**e**). The pressure distribution in a tablet was indicated by the distribution and agglomeration of crystal particles and divided into three sections (Section A: intermediate pressure, Section B: low pressure, Section C: high pressure. The enlarged part indicated the ultra-high pressure within the tablet with dense cluster of crystals of CLP I).

The different deformation behavior in the various regions of the tablets reflects the heterogeneous density distribution within the tablet. The degree of particle deformation reveals how the particles reacted to the applied pressures during tableting. The greater degree of deformation, the greater forces the particles experienced. By considering the different deformation behaviors of the CLP I or CLP II particles, the tablets can be segmented into three sections. The area subjected to the high pressure tended to be close to the surface, while intermediate pressure regions remained in the central core of the tablets; the low force region is the middle layer between the surface and the central core of the tablets. Figure 6(e) illustrates these sections of the pressure distribution spaces: section A as the intermediate pressure zone, section B as the lowest pressure area, and section C as the highest pressure section.

### Quantification of crystal deformation behavior in tablets

Similar to SR-μCT, the traditional X-ray computed tomography method as a non-destructive technique can also be used for the measurement of the density distribution within the tablets. It provides cross-sectional images in planes through a component based on the different absorptions of materials by X-ray. In comparison with traditional X-ray computed tomography, SR-μCT has more advantages with higher resolution, extensive degree of differentiation, thus it is possible to extract single particle for the visualization and quantitative characterization of the deformation behavior by SR-μCT. The shapes of particles varied with changes of 3D parameters accordingly during compaction. Conventional detection methods cannot generate a 3D quantitative analysis easily, whilst SR-μCT provides the *in situ* 3D parameters of each particle for further statistical analysis.

The volume distribution of the particles changed during tablet formation (Fig. 5, Table 1). Before tableting, most of the particle volumes of CLP I and CLP II were distributed in the same range from 4.0E + 06 μm^3^ to 1.2E + 07 μm^3^. After compaction, some particles were crushed and pressed into a large cluster. If the clusters were rejected, the particles volume distribution of CLP I mainly ranged from 3.2E + 06 μm^3^ to 4.0E + 06 μm^3^, while the particles volume distribution of CLP II ranged from 4.0E + 06 μm^3^ to 1.2E + 07 μm^3^. Generally, the particle volumes of both CLP I and CLP II were reduced. The different changes in the particles volume distribution reflected the difference in their deformation behaviors during tableting.

The sphericity distribution also changed after tableting (Fig. 5, Table 1). Before tableting, most particles were distributed from 0.85 to 0.95. The sphericity of a small percentage of particles that originally exhibited poor sphericity increased after compaction. This effect may be linked to the dispersing trend of particles. However, the sphericity of most particles in the tablet decreased. The degree of change in the sphericity was higher in CLP II compared to CLP I. The data in Table 1 show that the sphericity of particles after tableting was reduced, and the width of the distribution became wider. Sphericity of CLP II particles is also observed to show larger changes than for CLP I. In addition, the sphericity of CLP II particles was smaller than CLP I after tableting. This finding supports the view that particles of CLP II are more readily deformed, exhibiting greater compressibility; this trait is defined as the ability to be reduced in volume at a given pressure.

Ellipsoid parameters of individual crystal particles were also calculated (Fig. 5, Table 1). The comparison between the morphology before and after the compaction showed the detailed deformation behavior of single particle. Oblate and prolate described the degree for the shape of object deviates from an ideal sphere. When the oblate and prolate equal to zero, the object is a standard sphere. Higher values of the prolate parameters make the object like a “cigar”, while the increase of oblate makes the sphere flatter with a shape close to a “disc”. The value of Oblate/Prolate-1 was also evaluated as shown in Fig. 5 and Table 1. The oblate and prolate values of CLP I original crystal particles were both larger than that of CLP II, indicating the particle of CLP I had a longer axis and flatter shape in comparison with CLP II. After the compaction, the average value of oblate was increased and prolate was decreased for both CLP I and CLP II. The deformation during compaction made crystal particles flatter and the cracks happened against the longest axis. During the compaction, the oblate/prolate-1 value for both CLP I and CLP II changed consistently. The original oblate/prolate-1 value of CLP I was 0.354, which specified the flatter shape. In contrast, the value of -0.030 for CLP II was very close to 0, which specified the cylinder-like shape. After compaction, both the oblate/prolate-1 value of CLP I and CLP II were increased remarkably. For the CLP I, the increased oblate/prolate-1 even reached an average value of 1.616.

In combination with the result of volume change and height of tablet, the deformation process of CLP I and CLP II can be illustrated. Under the compression, the relative importance of three different deformation mechanisms dominates the consolidation of CLP I was Plastic ≥Elastic >Brittle Fracture. Theoretically, plastic deformation facilitates the formation of permanent particle–particle contact regions during compaction, the binding between particles has also been observed in Figs 4 and 6e. The superior mechanical properties could be owing to the higher density and strongest interaction between the molecules, resulting in the most favorable packing of molecules in the crystal^24^. In contrast, the sequence of CLP II was Brittle Fracture > Plastic > Elastic. This explains the good compressibility and higher densification of CLP II, for the crystal particles are fractured and introduce to the reduction in volume as a result of an applied pressure. Owing to the plastic deformation and binding between particles under compression, CLP I shows great tabletability and compactibility, which can be observed easily in Fig. 4^24^.

The sphericity, volume and position of particles in a tablet affect particle behavior on compaction (Fig. 6). This feature is clearly different between CLP I and CLP II. After tableting, the particles of CLP II exhibited a smaller volume and a lower sphericity than particles of CLP I in the same regions, because the CLP II particles fragmented more readily than those of CLP I. Nevertheless, the importance of particles position and sphericity of CLP II was consistent with CLP I behavior. The sphericity was reduced, and the volume was decreased, particularly in section C of the tablets.

In regions close to section C of the CLP I tablets, some small particles were observed due to the localized high pressure during compaction. The changes in sphericity were different in section A, section B and section C (Fig. 6 and Table 2). The sphericity of CLP I and CLP II were the highest in section B, and the sphericity was the smallest in section C. The distribution range for the sphericity in section C was wider than that for the two other areas.

The relationship between the changes in sphericity, volume and particle position inside the tablets showed that, the greater the pressure the particles were subject to, the greater was the change in sphericity and volume. Therefore, section C experienced high pressure, especially at the edges of the tablet, section A experienced the intermediate pressure, and section B experienced low pressure.

In this study, a new method combining synchrotron radiation X-ray microtomography and 3D reconstructions was developed to visualize and quantify the deformation behavior of clopidogrel bisulfate polymorphs CLP I and CLP II. At the same compression ratio, CLP II deformed easily with higher compressibility and hence higher densification as compared with CLP I. Both CLP I and CLP II particles exhibited different deformation behaviors in the different pressure distribution regions within the tablets. At the surface of the tablets, the degree of deformation behavior of particles was larger than other areas. The degree of deformation behavior was found to be linked by the changes of volume, sphericity and ellipsoid parameters. The different morphology of CLP I and CLP II particles showed different deformation behaviors after compaction. Plastic and elastic mechanisms dominated deformation behavior for CLP I have been observed. The disc shape like crystal particles got flatter under the pressure loading. Meanwhile the deformation of CLP II was dominated by the brittle fracture mechanism as the lower bonding strength cannot prevent the crystal particles from being crushed. The directly observation and quantitative characterization of deformation behavior were all in excellent agreement with the published results of mechanical test and molecular modeling^24^. SR-μCT is shown as a powerful tool to provide *in situ* 3D parameters of each particle inside tablets, to have an improved understanding of particle behavior after tableting.

## Materials and Methods

### Materials

Two polymorphs of CLP (CLP I and CLP II) were kindly donated by Shenyang Pharmaceutical University (Shenyang, Liaoning, China). Vinyl pyrrolidone and vinyl acetate copolymer (PVP/VA, Plasdone^®^ S630) was provided by Shanghai Chineway Pharmaceutical Tech Co., Ltd. (Shanghai, China) and used as a diluent. Gelatin capsules (2#, outer diameter is 5.97 ± 0.05 mm) were provided by Xinchang Hechang capsule and equipment Co. Ltd. (Zhejiang, China).

### Sample preparation

To observe the 3D structure of particles of the CLP polymorphs, samples composed of 5 mg of CLP particles (CLP I or II) and 10 mg PVP/VA were carefully mixed to ensure that the drug powders were evenly distributed within the diluent before filling the capsule used to hold the powders.

The tested tablets of CLP containing 25 mg of sieved (180–250 μm) CLP particles and 75 mg PVP/VA particles were prepared. The PVP/VA powder was sieved to obtain samples portions with an average size of 150 μm. Although CLP particles were sieved by the same mesh sieve, CLP particles may have different size due to their different morphology. A rotary tablet presser (ZP-5; Tianjiu Machinery factory, Shanghai, China) was equipped with 5 mm D-tooling with slightly concave-faced punch tips and a feed frame was used to fill the die uniformly. The position of the bottom punch was adjusted, and the nearest distance between the two punches was about 3,000 μm. During the compaction, the rotation speed was set at 8 rpm. These tablets were compacted under the same conditions to ensure that the compression ratio was the same, and the deformation behavior of different CLP particles was hardly contributing to tensile strength of the different tablets, but to the morphology of different particles. The powders for each tablet was weighed before tableting and was set as 100 ± 5 mg. The height of the acquisition window during SR-μCT scanning was 4.5 mm, covering the whole side of the tablet while performing the CT scan.

PVP/VA S630 was proven to be a good diluent for CLP particles in the tablets. By experimental verifications, when excipients like lactose, cornstarch, and microcrystalline were used, the CLP particles were easy to be compacted into large particles, which made it unable to detect CLP particles easily by SR-μCT.

### Powder X-ray diffraction

PXRD was recorded on a Bruker D8 Advance (Siemens) powder diffractometer. The measurement conditions were as follows: a 2.2 kW sealed Cu X-ray source, a graphite monochromator to filter out the Cu K β radiation and a NaI (T l) scintillation detector. The scans were performed between 3° and 40° 2θ with a 0.02° step size and a counting time of 0.1 s per step.

### Scanning electron microscopy

The SEM test was carried out with a Hitachi S520 device (Hitachi, Japan) at an accelerating voltage of 20 kV.

### SR-μCT

The SR-μCT tomographic images of prepared samples were acquired using the BL13W1 beam line at the Shanghai Synchrotron Radiation Facility (SSRF). X-rays were derived from an electron storage ring with an accelerated energy of 3.5 GeV, and an average beam current of 180 mA. The samples were scanned with photon energy of 16.0 keV. The size of the beam was approximately 45 mm (horizontal) ×5 mm (vertical) and a double-crystal monochromator with Si (111) and Si (311) crystals was used to monochromatize the X-rays. The monochromatized has a flux density about 5.8 × 10^10^ ph/s/mm^2^ and the energy resolution was ΔE/E = 5 × 10^−3^. After penetrating the sample, the X-rays were converted into visible light by a YAG:Ce scintillator (200 μm thickness). The projections were magnified by diffraction-limited microscope optics (2 × magnification) and digitized using a high-resolution 2,048 pixel × 2,048 pixel CCD camera with a physical pixel size of 7.4 μm (pco.2000, PCO AG, Kelheim, Germany). The effective pixel size was 3.7 μm, the exposure time was 2.0 s, and the sample-to-detector distance was 12 cm. For each acquisition, 720 projection images were captured with an angular step size of 0.15° for 180°. Flat-field and dark-field images were also collected during each acquisition procedure, in order to correct the electronic noise and variations in the X-ray source brightness.

The projected images were reconstructed using direct filtered back projection algorithm. To enhance the quality of reconstructed slices, the X-TRACT SSRF CWSx64 (Commonwealth Scientific and Industrial Research Organization, Australia, http://www.ts-imaging.net/Default.aspx) was used for phase contrast extraction. The 3D rendered data were analyzed with commercially available software (VGStudio Max (Version 2.1, Volume Graphics GmbH, Germany) and Image Pro Analyzer 3D (Version 7.0, Media Cybernetics, Inc., USA) to obtain the qualitative and quantitative data, respectively.

## Additional Information

**How to cite this article**: Yin, X.-Z. *et al.* Visualization and quantification of deformation behavior of clopidogrel bisulfate polymorphs during tableting. *Sci. Rep.* **6**, 21770; doi: 10.1038/srep21770 (2016).

## Figures and Tables

**Table 1:** Sphericity of CLP I and CLP II before and after tableting.

Parameters	CLP I	CLP II
Sphericity-Before tableting	0.873 ± 0.056	0.840 ± 0.045
Sphericity-After tableting	0.826 ± 0.089	0.747 ± 0.011
Oblate-Before tableting	0.435 ± 0.005	0.375 ± 0.005
Oblate-After tableting	0.467 ± 0.005	0.400 ± 0.007
Prolate-Before tableting	0.392 ± 0.005	0.449 ± 0.008
Prolate-After tableting	0.341 ± 0.004	0.406 ± 0.007
Eclipse-Before tableting	0.354 ± 0.040	−0.030 ± 0.032
Eclipse-After tableting	1.616 ± 0.050	0.695 ± 0.059

**Table 2:** Sphericity of CLP I and CLP II in section A (intermediate pressure), section B (low pressure) and section C (high pressure) regions.

Sphericity	CLP I	CLP II
Section A (intermediate pressure)	0.841 ± 0.099	0.754 ± 0.094
Section B (low pressure)	0.858 ± 0.067	0.767 ± 0.110
Section C (high pressure)	0.803 ± 0.096	0.735 ± 0.110

## References

[b1] Celik M. & Driscoll C. E. An overview of the effects of some physicochemical and mechanical characteristics of particulates on the compaction and post-compaction properties of compacts. Drug Develop Ind Pharm 19, 2119–2141 (1993).

[b2] Jain S. Mechanical properties of powders for compaction and tableting: an overview. Pharm Sci Technol Today 2, 20–31 (1999). 10234200 10.1016/s1461-5347(98)00111-4

[b3] Scholl J., Bonalumi D., Vicum L., Mazzotti M. & Muller M. *In situ* monitoring and modeling of the solvent-mediated polymorphic transformation of L-glutamic acid. Crystal Growth Des 6, 881–891 (2006).

[b4] Sun C. Q. & Grant D. J. W. Influence of crystal structure on the tableting properties of sulfamerazine polymorphs. Pharm Res 18, 274–280 (2001). 11442264 10.1023/a:1011038526805

[b5] Feng Y. S. & Grant D. J. W. Influence of crystal structure on the compaction properties of n-alkyl 4-hydroxybenzoate esters (Parabens). Pharm Res 23, 1608–1616 (2006). 16783478 10.1007/s11095-006-0275-9

[b6] Patel S., Kaushal A. M. & Bansal A. K. Compression physics in the formulation development of tablets. Crit Rev Ther Drug Carrier Syst 23, 1–65 (2006). 16749898 10.1615/critrevtherdrugcarriersyst.v23.i1.10

[b7] Busignies V. *et al.* Quantitative measurements of localized density variations in cylindrical tablets using X-ray microtomography. Eur J Pharm Biopharm 64, 38–50 (2006). 16621489 10.1016/j.ejpb.2006.02.007

[b8] Nebgen G., Gross D., Lehmann V. & Muller F. H-1-Nmr microscopy of tablets. J Pharm Sci 84, 283–291 (1995). 7616364 10.1002/jps.2600840304

[b9] Macleod H. M. & Marshall K. Determination of density distributions in ceramic compacts using autoradiography. Powder Technol 16, 107–122 (1977).

[b10] Sinka I. C., Burch S. F., Tweed J. H. & Cunningham J. C. Measurement of density variations in tablets using X-ray computed tomography. Int J Pharm 271, 215–224 (2004). 15129988 10.1016/j.ijpharm.2003.11.022

[b11] Upadhyay P., Khomane K. S., Kumar L. & Bansal A. K. Relationship between crystal structure and mechanical properties of ranitidine hydrochloride polymorphs. Crystengcomm 15, 3959–3964 (2013).

[b12] Joiris E., Di Martino P., Berneron C., Guyot-Hermann A. M. & Guyot J. C. Compression behavior of orthorhombic paracetamol. Pharm Res 15, 1122–1130 (1998). 9688070 10.1023/a:1011954800246

[b13] Yin X. Z. *et al.* Fractal structure determines controlled release kinetics of monolithic osmotic pump tablets. J Pharm Pharmacol 65, 953–959 (2013). 23738722 10.1111/jphp.12056

[b14] Li H. Y. *et al.* Microstructural investigation to the controlled release kinetics of monolith osmotic pump tablets via synchrotron radiation X-ray microtomography. Int J Pharm 427, 270–275 (2012). 22366382 10.1016/j.ijpharm.2012.02.017

[b15] Elmoutaouakkil A., Fuchs G., Bergounhon P., Peres R. & Peyrin F. Three-dimensional quantitative analysis of polymer foams from synchrotron radiation x-ray microtomography. J Phys D Appl Phys 36, A37–A43 (2003).

[b16] Weiss P. *et al.* Synchrotron X-ray microtomography (on a micron scale) provides three-dimensional imaging representation of bone ingrowth in calcium phosphate biomaterials. Biomaterials 24, 4591–4601 (2003). 12951002 10.1016/s0142-9612(03)00335-1

[b17] Tafforeau P. *et al.* Applications of X-ray synchrotron microtomography for non-destructive 3D studies of paleontological specimens. Appl Phy a-Mat Sci Process 83, 195–202 (2006).

[b18] Farber L., Tardos G. & Michaels J. N. Use of X-ray tomography to study the porosity and morphology of granules. Powder Technol 132, 57–63 (2003).

[b19] Crean B. *et al.* Elucidation of the internal physical and chemical microstructure of pharmaceutical granules using X-ray micro-computed tomography, Raman microscopy and infrared spectroscopy. Eur J Pharm Biopharm 76, 498–506 (2010). 20801216 10.1016/j.ejpb.2010.08.006

[b20] Yin X. Z. *et al.* Quantification of swelling and erosion in the controlled release of a poorly water-soluble drug using synchrotron X-ray computed microtomography. AAPS J 15, 1025–1034 (2013). 23861022 10.1208/s12248-013-9498-y PMC3787229

[b21] Chen L. *et al.* Identification of the polymorphs of clopidogrel bisulfate based on the steric morphology parameters of crystals. Yao Xue Xue Bao 48, 1459–1463 (2013). 24358781

[b22] Zupancic V., Kotar-Jordan B., Plevnik M., Smrkolj M. & Vrecer F. Similarity of solid state structures of R- and S-isomers of clopidogrel hydrogensulphate salt. Pharmazie 65, 389–390 (2010). 20503936

[b23] Uvarov V. & Popov I. Development and metrological characterization of quantitative X-ray diffraction phase analysis for the mixtures of clopidogrel bisulphate polymorphs. J Pharm Biomed Anal 46, 676–682 (2008). 18191358 10.1016/j.jpba.2007.11.026

[b24] Khomane K. S., More P. K. & Bansal A. K. Counterintuitive compaction behavior of clopidogrel bisulfate polymorphs. J Pharm Sci 101, 2408–2416 (2012). 22488254 10.1002/jps.23148

[b25] Eiliazadeh B., Briscoe B. J., Sheng Y. & Pitt K. Investigating density distributions for tablets of different geometry during the compaction of pharmaceuticals. Particulate Sci Technology 21, 303–316 (2003).

